# Acceptability and implementation of PhysioDirect telephone advice and treatment services: a multi-perspective

**Published:** 2012-06-15

**Authors:** Jennifer Pearson, Jane Richardson, Mike Calnan, Chris Salisbury, Nadine E Foster

**Affiliations:** Keele University, UK; Keele University, UK; University of Kent, UK; University of Bristol, UK; Keele University, UK

**Keywords:** telecare, PhysioDirect, physiotherapy, qualitative, musculoskeletal

## Abstract

**Introduction:**

In response to long waiting lists and problems with access to primary care physiotherapy, several Primary Care Trusts (PCTs) have developed physiotherapy-led telephone assessment and treatment services. The MRC funded PhysioDirect trial is a randomised trial in four PCTs with a total of 2252 patients comparing this approach with usual physiotherapy care, where patients join a waiting list for face-to-face physiotherapy.

**Aims:**

This nested qualitative study aimed to explore and understand the key issues that determine acceptability of PhysioDirect services from the perspectives of patients, physiotherapists and their managers, GPs and commissioners.

**Methods:**

Semi-structured interviews were conducted with 57 purposively sampled patients with musculoskeletal problems participating in the randomised trial. Sixteen physiotherapists, 4 physiotherapy managers, 8 GPs and 4 PCT commissioners were interviewed. The framework method was used to analyse the qualitative data.

**Results:**

All stakeholder groups perceived the PhysioDirect service as helpful in improving access to physiotherapy care by reducing physiotherapy waiting times. The physiotherapists and their managers perceived that physiotherapists could safely diagnose patients with musculoskeletal problems over the telephone. The GPs and commissioners raised concerns about the accuracy of diagnoses reached over the telephone and perceived it as a triage service which precedes face-to-face contact. Both patients and physiotherapists felt that the lack of visual information impaired their ability to effectively communicate their health problems over the telephone and both perceived that the PhysioDirect assessment was less personal than face-to-face contact. Patients expressed their concerns about trusting the expertise and knowledge of the physiotherapist without knowing them personally, with both patients and physiotherapists seeing the PhysioDirect service as impairing continuity of care. However, both patients and physiotherapists found that the PhysioDirect service worked particularly well as a medium to provide early, self-management advice. Physiotherapy managers found the unpredictable nature of the timing and volume of patient calls to the PhysioDirect service difficult to manage. Physiotherapy managers, GPs and commissioners had divergent views about the information needed to support future implementation of a PhysioDirect service. Service commissioners also appeared to have wide ranging and unrealistic expectations of the type of data that they wanted from physiotherapy managers in order to support decisions about commissioning PhysioDirect services.

**Conclusions:**

The PhysioDirect service was perceived by the patients, physiotherapists and their managers, as well as GP and commissioners as broadly acceptable. All three groups felt that the PhysioDirect service improved access to physiotherapy services. Both patients and physiotherapists had some concerns that the PhysioDirect service impaired the development of a good therapeutic relationship. The key challenges to future implementation of PhysioDirect services were managers’ ability to accurately allocate physiotherapy time to the service, along with providing the range of data that commissioners expected from a new service. Despite these reservations, all stakeholders could foresee PhysioDirect as one option of access for future physiotherapy services.

